# JTT-130, a Novel Intestine-Specific Inhibitor of Microsomal Triglyceride Transfer Protein, Improves Hyperglycemia and Dyslipidemia Independent of Suppression of Food Intake in Diabetic Rats

**DOI:** 10.1155/2014/803832

**Published:** 2014-05-07

**Authors:** Shohei Sakata, Makoto Ito, Yasuko Mera, Tomohiko Sasase, Hiromi Yamamoto, Makoto Kakutani, Takeshi Ohta

**Affiliations:** Biological/Pharmacological Research Laboratories, Central Pharmaceutical Research Institute, Japan Tobacco Inc., 1-1 Murasaki-cho, Takatsuki, Osaka 569-1125, Japan

## Abstract

We investigated the effects of JTT-130 on glucose and lipid metabolism independent of the suppression of feeding by comparing with pair-fed animals. Male Zucker diabetic fatty (ZDF) rats were divided into control, JTT-130 treatment, and pair-fed groups. The rats were fed with a regular powdered diet with or without JTT-130 as a food admixture for 6 weeks. We compared the effects on glucose and lipid metabolism in JTT-130 treatment group with those in pair-fed group. *Results*. Hyperglycemia in ZDF rats was prevented in both JTT-130 treatment and pair-fed groups, but the prevention in pair-fed group became poor with time. Moreover, reduction in plasma cholesterol levels was observed only in JTT-130 treatment group. JTT-130 treatment group showed improved glucose tolerance at 5 weeks after treatment and significant elevation of portal glucagon-like peptide-1 (GLP-1) levels. The hepatic lipid content in JTT-130 treatment group was decreased as compared with pair-fed group. Furthermore, pancreatic protection effects, such as an increase in pancreatic weight and an elevation of insulin-positive area in islets, were observed after JTT-130 treatment. *Conclusions*. JTT-130 improves hyperglycemia and dyslipidemia via a mechanism independent of suppression of food intake, which is ascribed to an enhancement of GLP-1 secretion and a reduction of lipotoxicity.

## 1. Introduction

Metabolic syndrome, which is characterized by visceral obesity, dyslipidemia, hyperglycemia, and hypertension, has become a major public health challenge all over the world [1]. The metabolic syndrome refers to the clustering of cardiovascular disease risk factors that are present in many people who are at increased risk for cardiovascular events and type 2 diabetes [2]. The cause of metabolic syndrome remains obscure, but there are many reports about the etiology. Reaven proposed that insulin resistance played a causative role [3], and Lemieux et al. suggested that visceral obesity and the hypertriglyceridemic waist phenotype are central components [4]. Therapies targeted to specific components of metabolic syndrome, such as glycemic control, management of dyslipidemia, and reduction of prothrombotic state, should help to minimize cardiovascular risk [2].

The microsomal triglyceride transfer protein (MTP) is known to take part in the mobilization and secretion of triglyceride- (TG-) rich lipoproteins from enterocytes and hepatocytes [5]. JTT-130, a novel intestine-specific inhibitor of MTP, suppresses the absorption of dietary fat and cholesterol in the intestine and decreases plasma triglyceride (TG) and total cholesterol (TC) levels [6]. JTT-130 suppresses food intake and gastric emptying with the elevation of plasma peptide YY (PYY) and glucagon-like peptide-1 (GLP-1) levels in a dietary fat-dependent manner [7]. Moreover, we showed that JTT-130 improves abnormalities of glucose and lipid metabolism in Zucker diabetic fatty (ZDF) rat or diet-induced obesity (DIO) rat with suppression of food intake [8, 9]. In this study, we prepared pair-fed rats in which food intake and diet pattern were matched to those in JTT-130-treated rats and investigated the effects on glucose and lipid metabolism independent of suppression of food intake in ZDF rats.

## 2. Materials and Methods

### 2.1. Materials

JTT-130, diethyl-2-({3-dimethylcarbamoyl-4-[(4′-trifluoromethylbiphenyl-2-carbonyl)amino]phenyl}acetyloxymethyl)-2-phenylmalonate, was synthesized by Japan Tobacco Inc. (Osaka, Japan). All other reagents used in this study were obtained commercially.

### 2.2. Animals and Diets

Male ZDF rats (ZDF/CrlCrlj-Lepr〈fa〉fatty) and lean rats (ZDF/CrlCrlj-Lepr〈fa〉lean) were obtained from Charles River Laboratories (Yokohama, Japan), individually housed with controlled temperature, humidity, and lighting (23 ± 3°C; 55 ± 15%; 12 h light/dark cycle, lights on at 8:00 AM), and provided with a powder diet (CRF-1; Oriental Yeast, Osaka, Japan) and water* ad libitum*. ZDF rats were divided into 3 groups: a control group, a JTT-130 treatment group, and a pair-fed group. ZDF rats in the JTT-130 treatment group were fed a powder diet mixed with an appropriate amount of JTT-130 (0.01-0.02%) to achieve a daily dose of approximately 10 mg/kg for 41 days, from 7 to 12 weeks of age. Food intake in JTT-130 treatment group was measured every 3 hours every day, using automatic food intake monitor and controller for rats (FDM-700AS; Melquest, Toyama, Japan). Food intake and dietary pattern in the pair-fed group were reproduced based on those in the JTT-130 treatment group measured on the previous day. A powder diet was fed individually, using the automatic measuring device mentioned above. Body weights were measured every 3 or 4 days during the experimental period. All procedures were conducted in accordance with the guidelines of the Japan Tobacco Animal Care Committee.

### 2.3. Measurement of Blood Chemical Parameters

Nonfasting plasma parameters, such as glucose, insulin, triglyceride (TG), total cholesterol (TC), and free fatty acid (FFA) levels, were examined every 7 days. Blood samples were collected from the tail vein. The glucose, TG, and TC levels were measured using commercial kits (Roche Diagnostics, Basel, Switzerland) and an automatic analyzer (Hitachi 7170S; Hitachi Ltd., Tokyo, Japan). Plasma insulin level was measured with a rat-insulin enzyme-linked immunosorbent assay (ELISA) kit (Morinaga Institute of Biological Science, Yokohama, Japan). Plasma FFA level was measured using NEFA *C*-test (Wako Pure Chemicals Industries Ltd., Osaka, Japan).

### 2.4. Oral Glucose Tolerance Test

Rats were fasted for 24 h and then administered orally with glucose (2 g/kg; Wako Pure Chemicals Industries Ltd., Osaka, Japan) on day 35 of treatment. Blood samples were collected from the tail vein just before glucose loading, and at 15, 30, 60, and 120 min after glucose loading. They were centrifuged to obtain plasma for the measurement of glucose and insulin. The homeostasis model assessment-insulin resistance (HOMA-IR) was calculated from fasting plasma glucose and insulin levels as follows: HOMA-IR = fasting plasma glucose (mg/dL) × fasting plasma insulin (ng/mL) × 26/405.

### 2.5. GLP-1 Level in the Portal Vein

Necropsy was performed on day 41 of treatment. The rats were anesthetized with diethyl ether, and blood samples were collected from the portal vein, transferred into tubes containing a DPP4 inhibitor (Millipore, Billerica, MA, USA) and aprotinin (Wako Pure Chemicals, Osaka, Japan) on ice, and centrifuged at 4°C, 10,000 ×g, for 5 min to obtain plasma. The concentration of GLP-1 in plasma was measured using a GLP-1 ELISA kit (Linco Research Inc.).

### 2.6. Measurement of Hepatic TG and TC Levels

The livers were removed from the rats and weighed. Liver fragments were homogenized in chloroform/methanol (2 : 1, vol/vol). A sample of the organic phase was collected, dried, and suspended in isopropyl alcohol. The extracted triglycerides and cholesterol were enzymatically determined using LiquiTech TGII and LiquiTech TCII (Roche Diagnostics K.K., Tokyo, Japan), respectively.

### 2.7. Immunohistochemical Evaluation of Pancreatic *β* Cells

Pancreas samples were weighed and fixed in 10% neutral buffered formalin and embedded in paraffin. Serial sections were prepared and stained with hematoxylin and eosin for light microscopic evaluation. Immunohistochemistry was performed on sections of pancreas with an anti-insulin antibody (Dako, Tokyo, Japan) by indirect staining using HRP-labeled anti-rabbit IgG (Dako). Total insulin-positive (*β* cell) area and total pancreas area were imaged with fluorescence microscope (BZ-9000, Keyence, Itasca, IL, USA), and proportion of insulin-positive area (%) was calculated.

### 2.8. Statistical Analysis

Data were expressed as mean values ± s.d or + s.d. *F*-tests were conducted for testing the equality of variances. If the variances were found to be equal, Student's *t*-test was then conducted. If the variances were found to be unequal, Welch's *t*-test was then conducted. *P* < 0.05 was considered statistically significant.

## 3. Results

### 3.1. Food Intake and Body Weights

**Figure 1:**
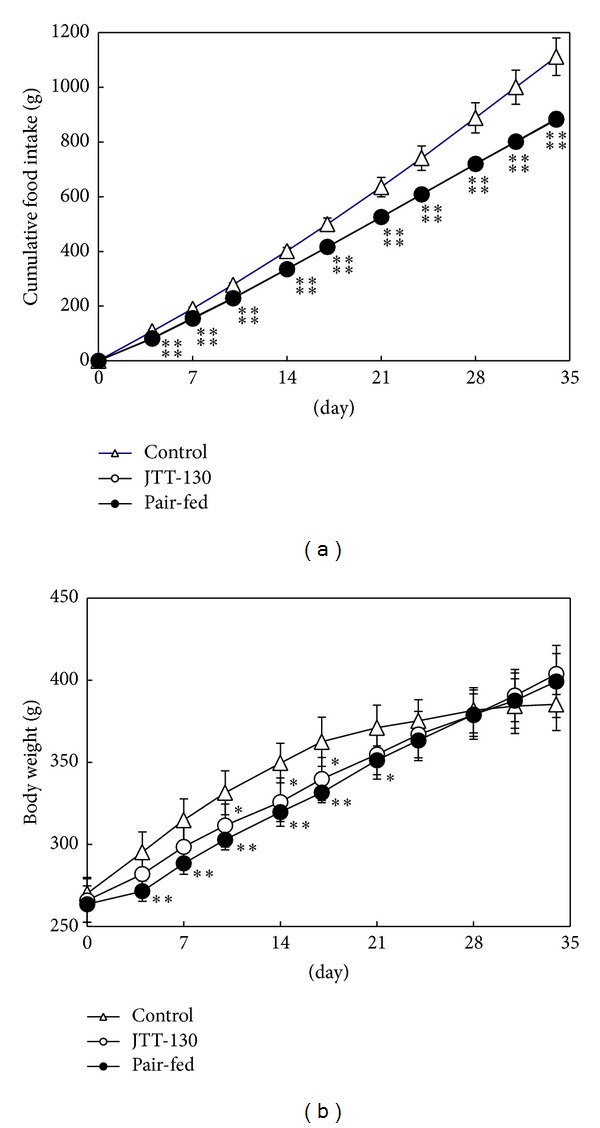
Changes in food intake (a) and body weight (b) in control group, JTT-130 treatment group, and pair-fed group. Data represent mean values ± s.d. (*n* = 5-6). *P < 0.05, **P < 0.01: significantly different from control group.

Changes in cumulative food intake and body weight are shown in Figure 1. The cumulative food intakes in JTT-130 treatment group were decreased after day 4 of treatment as compared with those in control group, and the decrease of food intake was sustained throughout the experimental period (on day 34, control group, 1111.3 ± 68.1 g; JTT-130 treatment group, 884.1 ± 24.6 g) (Figure 1(a)). The body weights in JTT-130 treatment group were significantly decreased from day 10 to day 17 of treatment, as compared with those in control group. At the later period of the experiment, the body weight gains in the control group decreased because of the development of diabetes, so the body weights in the JTT-130 treatment group inversely became higher than those in control group. The body weights in pair-fed group also were decreased, from day 4 to day 21 of treatment, as compared with those in control group, but there were no differences in body weights between JTT-130 treatment group and pair-fed group (Figure 1(b)).

### 3.2. Blood Chemical Parameters

**Figure 2:**
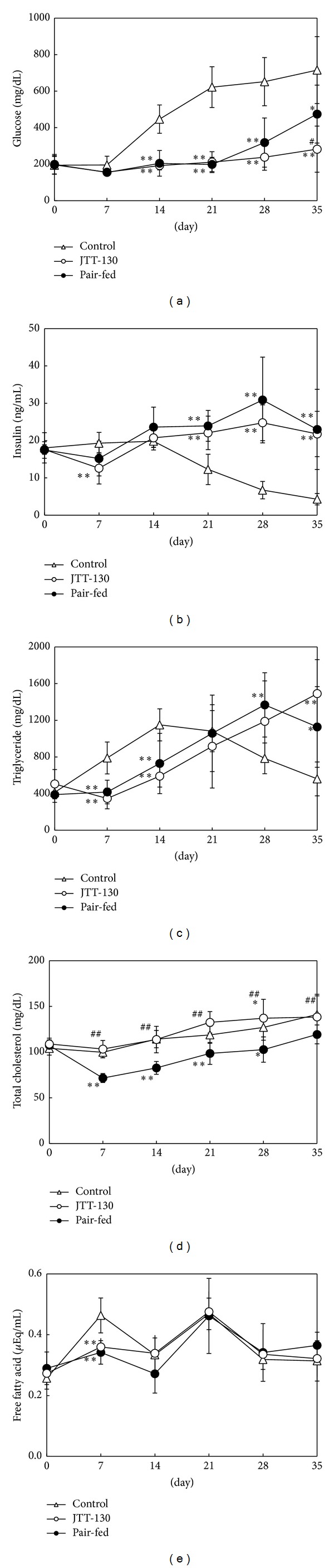
Changes in blood glucose (a), insulin (b), triglyceride (c), total cholesterol (d), and free fatty acid (e) levels in control group, JTT-130 treatment group, and pair-fed group. Data represent mean values ± s.d. (*n* = 5-6). *P < 0.05, **P < 0.01: significantly different from control group. ^#^ *P* < 0.05, ^##^ *P* < 0.01: significantly different from pair-fed group.

Changes in blood chemical parameters, such as glucose, insulin, TG, TC, and FFA levels, are shown in Figure 2. It is well known that ZDF rats exhibit severe hyperglycemia and decreased insulin level along with the exhaustion of pancreatic beta cells [10]. Also in this study, plasma glucose levels in control group increased after day 14 of treatment, resulting in severe hyperglycemia. On the other hand, the hyperglycemia was clearly prevented in both JTT-130 treatment and pair-fed groups. After day 28 of treatment, plasma glucose levels in the pair-fed group became higher than those in the JTT-130 treatment group and were significantly increased on day 35 of treatment (control group, 715 ± 183 mg/dL; JTT-130 treatment group, 282 ± 127 mg/dL; pair-fed group, 474 ± 159 mg/dL) (Figure 2(a)). Along with hyperglycemia, plasma insulin levels in control group decreased gradually after day 21 of treatment with aging, suggesting the exhaustion of pancreatic beta cells. Plasma insulin levels in pair-fed and JTT-130 treatment groups at day 7 of treatment tended to decrease or decreased as compared with those in control group, and after day 21 of treatment, the plasma insulin levels in both groups were inversely higher than those in control group. There were no differences in plasma insulin levels between JTT-130 treatment group and pair-fed group (Figure 2(b)).

Plasma TG levels in JTT-130 treatment and pair-fed groups were lower at days 7 and 14 of treatment, as compared with those in control group, and the TG levels in JTT-130 treatment and pair-fed groups significantly increased after day 28 (Figure 2(c)). There were no differences in plasma TG levels between JTT-130 treatment group and pair-fed group. Plasma TC levels in JTT-130 treatment group were significantly decreased after day 7 of treatment, as compared with those in control group, but the TC levels in pair-fed group were comparable to those in control group during the experimental period (Figure 2(d)). Plasma FFA levels in JTT-130 treatment and pair-fed groups significantly decreased at day 7 of treatment; however, after day 14 of treatment, there were no differences among the 3 groups (Figure 2(e)).

### 3.3. Oral Glucose Tolerance Test

**Figure 3:**
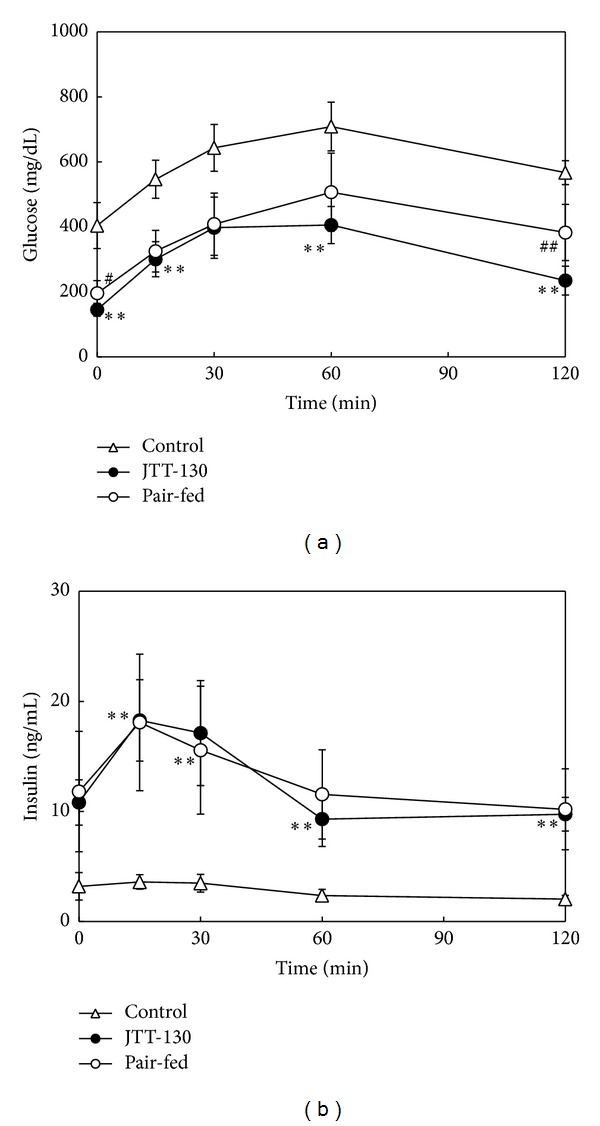
Changes in blood glucose (a) and insulin (b) levels in glucose-loaded ZDF rats. An oral glucose tolerance test was performed on day 35 of treatment. Data represent mean values ± s.d. (*n* = 5-6). *P < 0.05: significantly different from control group.

Plasma glucose levels before and after glucose loading were significantly decreased in JTT-130 treatment group, as compared with those in control group, and JTT-130 treatment showed a significant improvement of glucose tolerance. In pair-fed group, plasma glucose levels before and after glucose loading tended to decrease, but the glucose levels before and at 120 min after glucose loading were significantly higher than those in JTT-130 treatment group (Figure 3(a)). Plasma insulin levels in the glucose tolerance test were significantly elevated in JTT-130 treatment group, as compared with those in control group, but there were no differences between JTT-130 treatment group and pair-fed group (Figure 3(b)). The HOMA-IR value tended to decrease in JTT-130 treatment group, as compared with that in pair-fed group (control group, 84.8 ± 16.6; JTT-130 treatment group, 101.2 ± 10.5; pair-fed group, 140.5 ± 22.1).

### 3.4. GLP-1 Level in Portal Vein

**Figure 4:**
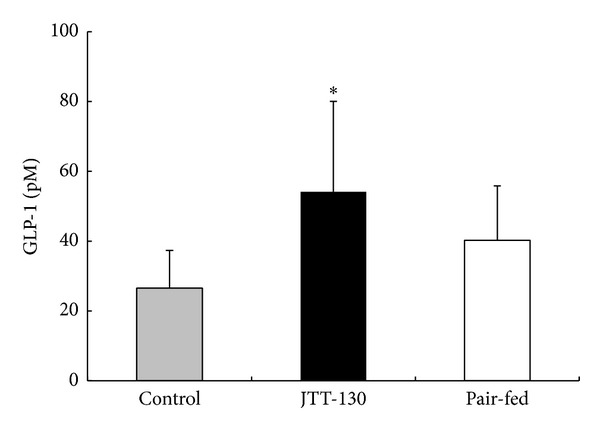
Portal GLP-1 levels in control group, JTT-130 treatment group, and pair-fed group. Necropsy was performed on day 41 of treatment and the GLP-1 levels were measured. Data represent mean values + s.d. (*n* = 5-6). *P < 0.05: significantly different from control group.

GLP-1 levels in portal vein were significantly elevated in JTT-130 treatment group, as compared with those in control group. The GLP-1 levels in pair-fed group tended to increase as compared with those in control group (control group, 26.5 ± 10.8 mg/dL; JTT-130 treatment group, 54.1 ± 26.0 mg/dL; pair-fed group, 40.3 ± 15.5 mg/dL) (Figure 4).

### 3.5. Hepatic Lipid Levels

**Figure 5:**
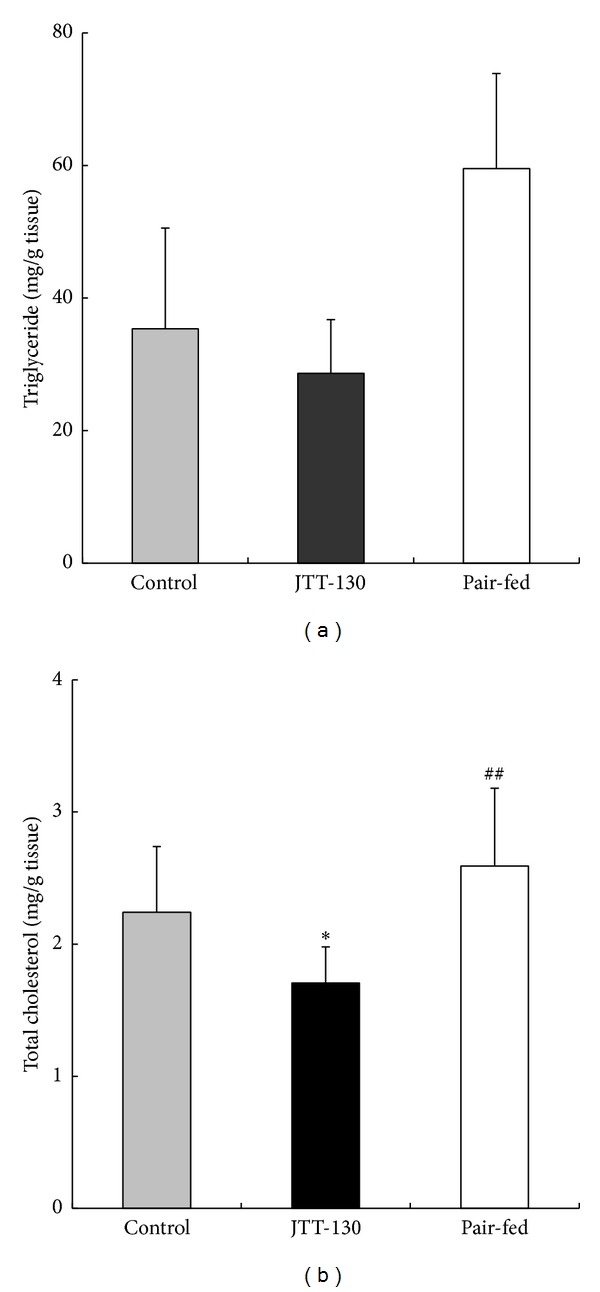
Hepatic triglyceride (a) and total cholesterol (b) levels in control group, JTT-130 treatment group, and pair-fed group. Necropsy was performed on day 41 of treatment and the lipid levels were measured. Data represent mean values + s.d. (*n* = 5-6). *P < 0.05: significantly different from control group. ^##^ *P* < 0.01: significantly different from pair-fed group.

Hepatic TG and TC contents were measured at necropsy (Figure 5). Hepatic TC contents in JTT-130 treatment group were significantly decreased as compared with those in control group (Figure 5(b)). However, hepatic TG and TC levels in pair-fed group were significantly higher than those in JTT-130 treatment group (Figure 5).

### 3.6. Pancreatic Analysis

**Figure 6:**
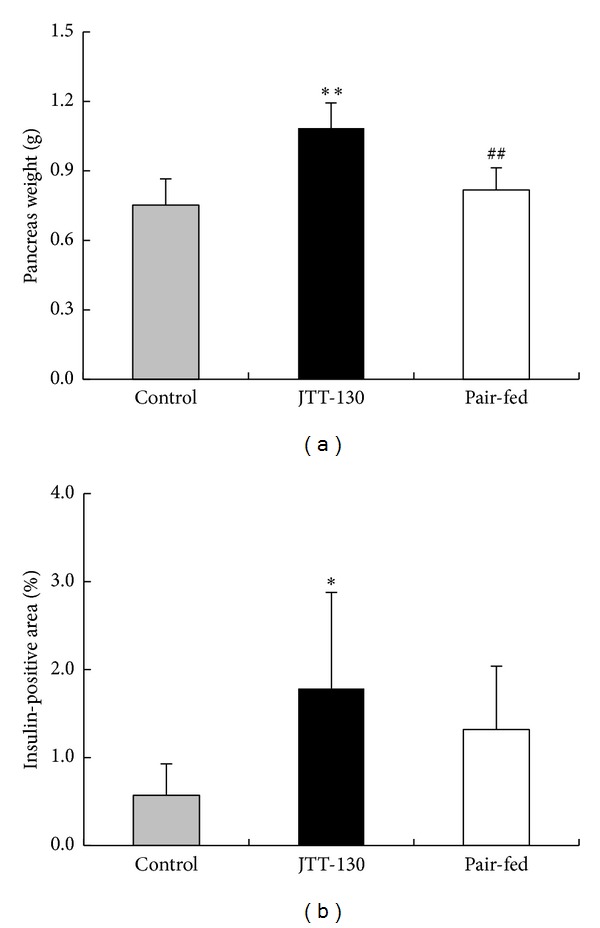
Pancreatic weight (a) and insulin-positive area in islets (b) in control group, JTT-130 treatment group, and pair-fed group. Data represent mean values + s.d. (*n* = 5-6). *P < 0.05, **P < 0.01: significantly different from control group. ^##^ *P* < 0.01: significantly different from pair-fed group.

Pancreatic weights in JTT-130 treatment group were significantly elevated, as compared with those in control group or in pair-fed group, but the weights in pair-fed group were comparable to those in control group (Figure 6(a)). Insulin-positive areas in islets in JTT-130 treatment group were also significantly elevated, as compared with those in control group (Figure 6(b)). The areas in pair-fed group tended to be increased as compared with control group, but not significantly.

## 4. Discussion

JTT-130 is an intestine-specific MTP inhibitor designed to be rapidly hydrolyzed to inactive metabolites immediately after its absorption in the intestine to avoid inhibition of hepatic MTP resulting in hepatic steatosis [6]. We previously reported that JTT-130 suppressed cumulative food intake along with the increased GLP-1 and PYY levels and ameliorated diet-induced obesity and glucose intolerance in Sprague-Dawley rats fed a 35% fat diet. JTT-130 increased the contents of triglycerides and free fatty acids in the intestinal lumen, which might contribute to the elevation of PYY and GLP-1 levels [7, 9]. We also reported that JTT-130 decreased the food intake and ameliorated the impaired glucose and lipid metabolism in ZDF rats [8]. While we already showed the antidiabetic effects of JTT-130 with suppressed food intake as described above, we had not examined the effects independent of its suppression of food intake. Since GLP-1 is primarily an antidiabetic peptide and inhibition of TG absorption in the intestine might lead to the diminished lipotoxicity, we expected JTT-130 possibly to exhibit antidiabetic effect via a mechanism other than suppression of food intake. We therefore investigated the effects of JTT-130 on glucose and lipid metabolism by means of a pair-feeding system in ZDF rats in the present study.

Food intake in the JTT-130 treatment group was measured every 3 hours every day and the food intake and dietary pattern were precisely reproduced in the pair-fed group. JTT-130 treatment showed a suppression of food intake (Figure 1(a)) as reported previously [8]. Body weight in the JTT-130 treatment and pair-fed groups showed similar change throughout the experimental period (Figure 1(b)). The body weight gain in the control group gradually decreased with severity of diabetes condition.

Hyperglycemia was prevented at days 14 and 21 in both JTT-130 treatment and pair-fed groups, but the inhibition in the pair-fed group became poor at the later period of experiment. On day 35, JTT-130 clearly prevented development of diabetes as compared with pair-feeding (Figure 2(a)). Besides these results, the improvement of glucose tolerance in the JTT-130 treatment group was more significant than that in the pair-fed group (Figure 3(a)) and the HOMA-IR was also improved.

The better glycemic control in JTT-130 treatment than pair-feeding is considered to be ascribable to enhanced insulin sensitivity, since there were no significant differences in the plasma insulin levels or the insulin secretion in glucose tolerance test between the two groups (Figure 3(b)). Hepatic lipid contents in pair-fed rats were elevated compared with those in control group (Figure 5). Since plasma insulin levels in pair-fed group were higher than those in control group (Figure 2(b)), the hepatic lipid levels are considered to be elevated in pair-fed rats. In this study, JTT-130 significantly decreased hepatic TG contents as compared with pair-feeding (Figure 5(a)). It is well known that ectopic lipid accumulation in nonadipocyte cells causes lipotoxic insults, including insulin resistance, apoptosis, and inflammation, and, especially, hepatic TG contents are associated with insulin resistance in humans [11, 12]. Brief calorie restriction and minimal weight loss primarily decreased intrahepatic TG content associated with increased hepatic insulin sensitivity [13]. Since JTT-130 directly suppresses TG absorption in small intestine, JTT-130 reduced hepatic TG contents, which might result in improved insulin sensitivity in the liver. In addition to this, the effects of JTT-130 on insulin sensitivity might be ascribed to the elevation of GLP-1. It is reported that the inhibition of intestinal MTP by JTT-130 leads to the elevation of lipid levels in the intestinal tissue and the following increase in blood GLP-1 levels [7]. The significantly increased level of portal GLP-1 was observed only in the JTT-130 treatment group (Figure 4). GLP-1 is known as one of the hypoglycemic hormone [14], while it is also reported that GLP-1 shows improvement of insulin sensitivity [15, 16]. Insulin-sensitizing effects have been observed with GLP-1 analogues in type 2 diabetic animal models. Exendin-4 treatment improved whole insulin sensitivity in ZDF rats, measured by increases in glucose infusion rate in a hyperinsulinemic euglycemic clamp [17]. In obese Zucker rats, 6 weeks of treatment with exenatide improved insulin sensitivity measured by insulin sensitivity index during a hyperinsulinemic euglycemic clamp [18]. Furthermore, Lee et al. suggested that GLP-1 reduces macrophage infiltration and directly inhibits inflammatory pathways in adipocyte and adipose tissue macrophages, possibly contributing to the improvement of insulin sensitivity [19]. It is important to determine intramuscular lipid levels in JTT-130 treatment as peripheral insulin sensitivity, as well as hepatic lipids, in further study.

In addition, GLP-1 is known to be able to prevent apoptosis in the pancreatic *β* cell and stimulate the proliferation and differentiation of insulin-secreting *β* cells in ZDF rats [20, 21]. In the present study, the pancreatic weight in ZDF rats significantly increased in the JTT-130 treatment group (Figure 6(a)), and the insulin-positive area in islets was also elevated by JTT-130 treatment (Figure 6(b)). The pancreatic protection effects with JTT-130 treatment in the rats might be caused by an increase in GLP-1 level. It is also reported that the pancreatic beta cells in ZDF rats have higher levels of TG contents than those in lean littermates [22], resulting in impaired glucose-stimulated insulin secretion. JTT-130 suppressed TG absorption in small intestine, so it may have favorable influence on pancreatic beta cell function through the removal of lipotoxicity.

Also, the JTT-130 treatment significantly reduced plasma TC level as compared with pair-feeding (Figure 2(d)). Since JTT-130 directly suppresses the absorption of dietary fat and cholesterol in the intestine [5, 6], the effects of JTT-130 on the reduction of hepatic TC contents are considered to be more significant than that of pair-feeding.

In summary, JTT-130-treated rats showed improved glucose and lipid abnormalities more significantly than pair-fed rats, accompanied with elevated portal level of GLP-1 and reduction of hepatic lipid content. These results suggest that JTT-130 improves the hyperglycemia and dyslipidemia independent of suppression of food intake by an enhancement of GLP-1 secretion and a reduction of lipotoxicity.

## References

[1] Eckel RH, Grundy SM, Zimmet PZ (2005). The metabolic syndrome. *The Lancet*.

[2] Wyne KL (2006). The metabolic syndrome: evolving evidence that thiazolidinediones provide rational therapy. *Diabetes, Obesity and Metabolism*.

[3] Reaven GM (1988). Role of insulin resistance in human disease. *Diabetes*.

[4] Lemieux I, Pascot A, Couillard C (2000). Hypertriglyceridemic waist: a marker of the atherogenic metabolic triad (hyperinsulinemia; hyperapolipoprotein B; small, dense LDL) in men?. *Circulation*.

[5] Xie Y, Newberry EP, Young SG (2006). Compensatory increase in hepatic lipogenesis in mice with conditional intestine-specific Mttp deficiency. *Journal of Biological Chemistry*.

[6] Mera Y, Odani N, Kawai T (2011). Pharmacological characterization of diethyl-2-({3-dimethylcarbamoyl-4-[(4′-trifluoromethylbiphenyl-2-carbonyl)amino]phenyl}acetyloxymethyl)-2-phenylmalonate (JTT-130), an intestine-specific inhibitor of microsomal triglyceride transfer protein. *Journal of Pharmacology and Experimental Therapeutics*.

[7] Hata T, Mera Y, Ishii Y (2011). JTT-130, a novel intestine-specific inhibitor of microsomal triglyceride transfer protein, suppresses food intake and gastric emptying with the elevation of plasma peptide YY and glucagon-like peptide-1 in a dietary fat-dependent manner. *Journal of Pharmacology and Experimental Therapeutics*.

[8] Hata T, Mera Y, Kawai T (2011). JTT-130, a novel intestine-specific inhibitor of microsomal triglyceride transfer protein, ameliorates impaired glucose and lipid metabolism in Zucker diabetic fatty rats. *Diabetes, Obesity and Metabolism*.

[9] Hata T, Mera Y, Tadaki H (2011). JTT-130, a novel intestine-specific inhibitor of microsomal triglyceride transfer protein, suppresses high fat diet-induced obesity and glucose intolerance in Sprague-Dawley rats. *Diabetes, Obesity and Metabolism*.

[10] Unger RH (1995). Lipotoxicity in the pathogenesis of obesity-dependent NIDDM: Genetic and clinical implications. *Diabetes*.

[11] Capeau J (2008). Insulin resistance and steatosis in humans. *Diabetes & Metabolism*.

[12] Virtue S, Vidal-Puig A (2010). Adipose tissue expandability, lipotoxicity and the Metabolic Syndrome—an allostatic perspective. *Biochimica et Biophysica Acta*.

[13] Kirk E, Reeds DN, Finck BN, Mayurranjan MS, Patterson BW, Klein S (2009). Dietary fat and carbohydrates differentially alter insulin sensitivity during caloric restriction. *Gastroenterology*.

[14] Drucker DJ (2007). The role of gut hormones in glucose homeostasis. *Journal of Clinical Investigation*.

[15] He M, Su H, Gao W (2010). Reversal of obesity and insulin resistance by a non- peptidic glucagon-like peptide-1 receptor agonist in diet-induced obese mice. *PLoS ONE*.

[16] Sebokova E, Bénardeau A, Sprecher U, Sewing S, Tobalina L, Migliorini C (2010). Taspoglutide, a novel human once-weekly analogue of glucagon-like peptide-1, improves glucose homeostasis and body weight in the Zucker diabetic fatty rat. *Diabetes, Obesity and Metabolism*.

[17] Young AA, Gedulin BR, Bhavsar S (1999). Glucose-lowering and insulin-sensitizing actions of exendin-4: studies in obese diabetic (*ob/ob, db/db*) mice, diabetic fatty Zucker rats, and diabetic rhesus monkeys (*Macaca mulatta*). *Diabetes*.

[18] Gedulin BR, Nikoulina SE, Smith PA (2005). Exenatide (exendin-4) improves insulin sensitivity and *β*-cell mass in insulin-resistant obese fa/fa Zucker rats independent of glycemia and body weight. *Endocrinology*.

[19] Lee YS, Park MS, Choung JS (2012). Glucagon-like peptide-1 inhibits adipose tissue macrophage infiltration and inflammation in an obese mouse model of diabetes. *Diabetologia*.

[20] Farilla L, Hongxiang H, Bertolotto C (2002). Glucagon-like peptide-1 promotes islet cell growth and inhibits apoptosis in Zucker diabetic rats. *Endocrinology*.

[21] Parsons GB, Souza DW, Wu H (2007). Ectopic expression of glucagon-like peptide 1 for gene therapy of type II diabetes. *Gene Therapy*.

[22] Lee Y, Hirose H, Ohneda M, Johnson JH, McGarry JD, Unger RH (1994). *β*-Cell lipotoxicity in the pathogenesis of non-insulin-dependent diabetes mellitus of obese rats: impairment in adipocyte-*β*-cell relationships. *Proceedings of the National Academy of Sciences of the United States of America*.

